# Association of interleukin-4, interleukin-13 gene polymorphisms, HLA-DQ and DR genotypes with genetic susceptibility of type-1 Diabetes Mellitus in Kuwaiti children

**DOI:** 10.3389/fped.2023.1118137

**Published:** 2023-04-06

**Authors:** Mohammad Z. Haider, Maysoun Al Rushood, Hind Alsharhan, Majedah A. Rasoul, Maria Al-Mahdi, Hessa Al-Kandari

**Affiliations:** ^1^ Department of Pediatrics, College of Medicine, Kuwait University, Jabriya, Kuwait; ^2^ Department of Pediatrics, Mubarak Al-Kabeer Hospital, Jabriya, Kuwait; ^3^ Department of Pediatrics, Farwaniya Hospital, Farwaniya, Kuwait; ^4^ Department of Pediatrics, Al-Adan Hospital, Adan, Kuwait; ^5^ Department of Population Health, Dasman Diabetes Institute, Dasman, Kuwait

**Keywords:** cytokine gene, HLA, polymorphism, type 1 diabetes mellitus, Kuwait

## Abstract

**Background:**

Type-1 diabetes mellitus (T1DM) is a complex multifactorial disease with an autoimmune etiology and is thought to result from an interaction between genetic and non-genetic factors. Cytokines play a crucial role in the pathogenesis of autoimmune diseases due to their effector and regulatory functions in immune responses. Interleukin-4 (IL4) and Interleukin-13 (IL13) are anti-inflammatory cytokines and are considered as important mediators in pathology of the autoimmune diseases.

**Methods:**

We have determined the genotype frequency of *IL4* gene promoter polymorphism (−590C/T, rs2243250), *IL13* gene polymorphism *p*.(Arg130Glu, rs20541) and human leukocyte antigen, HLA-DQ and DR genotypes in Kuwaiti children with T1DM to investigate their role in genetic susceptibility. This study included 261 Kuwaiti children with T1DM and 214 healthy controls. The genotypes for *IL4* (−590C/T) and *IL13 p*.(Arg130Glu) gene polymorphisms were detected by PCR-RFLP methods. HLA-DQ and DR genotypes were determined by sequence-specific PCR methods.

**Results:**

The CC genotype of *IL4* gene polymorphism (−590C/T) was significantly related to the risk for T1DM in Kuwaiti patients (OR 1.64). The homozygous AA (QQ) and heterozygous AG (RQ) genotypes of *IL13* gene polymorphism *p*.(Arg130Glu), also manifested a statistically significant association with T1DM (OR 2.92 and 4.79). In 55% T1DM patients, the HLA genotype was either DQ2/DQ2 or in combination with a DQ8 allele. Collectively, 91% Kuwaiti T1DM patients had either DQ2 or DQ8 alleles in different combinations highlighting them as the high risk-genotypes in comparison to the controls. In the case of HLA-DR, the genotypes DR3/DRB5, DR3/DR4, DR3/DR7 and DR4/DR4 showed highest frequency amongst the Kuwaiti T1DM patients and thus can be considered as high-risk genotypes when compared to the controls. A high degree of co-inheritance (>80%) was detected between *IL4* and *IL13* gene polymorphism genotypes (CC and QQ) and the high-risk HLA-DQ and DR genotypes amongst the Kuwaiti T1DM patients.

**Conclusions:**

We have identified the association of *IL4* and *IL13* gene polymorphisms with susceptibility to T1DM in Kuwaiti children and the co-inheritance of these polymorphisms with high-risk HLA genotypes. The findings may contribute to early identification of childhood diabetes.

## Introduction

Type-1 diabetes mellitus (T1DM) is an auto-immune, multi-factorial disease which is thought to result from T cell-mediated destruction of the pancreatic beta cells (1). It is considered to be caused by an interaction between the genetic and environmental factors (2). T1DM accounts for 80%–90% of diabetic children and adolescents (3–5). Several susceptibility loci involved in disease development have been identified and were consistently replicated in independent populations (6). The major histocompatibility complex (MHC) class II, the cytotoxic T-lymphocyte–associated antigen 4 (*CTLA4*) and the protein tyrosine phosphatase non-receptor type 22 (*PTPN22*) loci have all been proven important in the pathogenesis of autoimmunity globally, whereas insulin (*INS*) gene is a disease-specific T1DM susceptibility locus (6). Most of the susceptibility loci identified in the recent years have a clear role in modulation of T cell development and activation, indicating that common biological pathways may be implicated in the etiology of different autoimmune diseases (7). A number of recently published reviews and reports provided comprehensive information about the genetic risk factors (MHC and non-MHC loci), epidemiological aspects and immune system involvement in T1DM patients from Kuwait (8–11). These reports include information on genetic factors which influence both susceptibility and resistance to T1DM (8). It has also been reported that combination of genes linked to the MHC (major histocompatibility complex) have a major effect on risk of T1DM in addition to the known potent effects of HLA DQ and DR alleles/genotypes in different populations (8, 11).

Cytokines are low molecular weight extracellular proteins which act as immune response mediators. They are part of highly complex pathways which regulate the inflammatory process and are considered essential in manifesting response at the lesion site. In the pathogenesis of T1DM, stimulation of the innate immune system has been shown to play an important role (12). Previous reports have implicated pro-and anti-inflammatory cytokines in the events leading to the onset of diabetes mellitus, [DM] (13). It has been shown that definite pro and/or anti-inflammatory cytokines interfere with insulin responsive glucose uptake and stimulate insulin resistance in DM (13, 14). Cytokines play a crucial role in pathogenesis of several auto-immune diseases, and this is thought to be due to their effector and regulatory functions in immune and inflammatory processes (14). IL4, a critical anti-inflammatory cytokine and it has been implicated in the pathogenesis, activity and severity of various autoimmune diseases (15). A polymorphism (−C590T, rs2243250), in the promoter region of *IL4* gene has been suggested as one of the genetic risk factors in autoimmune diseases. Several previous reports have explored the relationship between this single nucleotide polymorphism (SNP) and genetic susceptibility of autoimmune diseases in different populations but the results from these studies remain inconclusive (15, 16).

Interleukin 13 (IL13) is an anti-inflammatory cytokine that is produced predominantly by CD4+ T cells with Th2 characteristics (17). IL13 plays an important role in the pathogenesis of Th2-mediated diseases (18). The human *IL13* gene is located on chromosome 5q31, approximately 12 kb upstream from the *IL4* gene (encoding for another Th2 cytokine). It has been proposed that IL13 downregulates the inflammatory process by suppressing the production of Th1 cytokines (19).

Association studies focusing on candidate genes involved in immune responses, such as those encoding elements of the T cell activation pathway, represent a useful approach for finding the T1DM susceptibility genes. IL4 and IL13 have been shown to protect against the disease development in rodent models of T1DM (20), suggesting the possibility that cytokines involved in the Th1 and/or Th2 pathways may play a significant role in T1DM pathogenesis (21). IL4 and IL13 are key components in the induction of immune responses and in downregulation of the Th1 lymphocyte phenotype. In humans, IL4 transcript levels have been shown to be greatly reduced in new-onset T1DM (22), and it has been speculated that IL13, as an anti-inflammatory cytokine and a mediator of the Th2 pathway represents a potential therapeutic approach in prevention of the T1DM (23–26). A number of studies have investigated the association between this polymorphism and the susceptibility of autoimmune diseases e. g. rheumatoid arthritis (RA), Graves' disease (GD), multiple sclerosis (MS) and systemic lupus erythematosus (SLE) (15). However, the findings from these studies remain inconsistent, mainly because of factors like small population size studied, inadequate statistical power and population variation (15). The involvement of *IL4* and *IL13* gene polymorphisms in a number of autoimmune diseases prompted us to investigate their role along with HLA-DQ and DR genotypes in genetic susceptibility of T1DM in a completely different population (Kuwaiti Arab children) which has a high incidence rate of T1DM.

## Materials and methods

In this study, we included Kuwaiti children diagnosed with T1DM from three major secondary and tertiary hospitals located in three different regions of Kuwait (Mubarak Al-Kabeer Hospital, Al-Adan Hospital and Farwaniya Hospital). The inclusion criteria used for T1DM patients was based on combined recommendations of the WHO Multinational Project for Childhood Diabetes (DiaMond Project) and the ISPAD Guidelines—International Society for Pediatric and Adolescent Diabetes Clinical Practice Consensus Guidelines 2014 (27):

(i) Diagnosis by a physician as diabetic
(ii) Placed on a daily dose of insulin before the 15th birthday
(iii) A Kuwaiti national resident in the area at the time of the first insulin administration.

The ISPAD Guidelines (27) provided detailed criteria for diagnosis of T1DM and these guidelines were strictly followed in this report. The controls were chosen very carefully and were un-related to the T1DM patients. The controls were evaluated by a trained diabetes specialist. They were fully healthy (without any immune system or another disease at the time of study), recruited randomly and all were above the age of 15 years. The age of the controls (above 15 years) was in accordance with recommendation of the WHO-DiaMond protocol, in order to make sure that the individuals chosen as controls had lived through the high-risk period (0–15 years) the time in which T1DM is most likely to develop.

The glycemic status in all the study subjects (T1DM patients and controls) was ascertained by determining the HbA1c levels. All the control subjects had their HbA1C below 5.7% in accordance with the ISPAD Guidelines (27). The clinical data was collected and recorded at the time of routine visits to the clinics by the T1DM patients. It included data on age, gender, age at the time of onset, levels of glycemic control (HbA1c values etc.) and the demographic information.

### Genotyping

Total genomic DNA was extracted from the peripheral leukocytes using a standard method (28). The genotypes were detected by PCR-RFLP (polymerase chain reaction-restriction fragment length polymorphism) methods descried below:

### *IL4* gene (-C590T; rs2243250) promoter polymorphism

The primers and procedures used for determination of *IL4* gene -C590T, rs2243250 promoter polymorphism, have been described earlier (29). The C→T transition at codon 590 of the *IL4* gene promoter region, abolished a restriction site for *Bsm*F1 in the T-allele. The polymorphism was detected by *Bsm*F1 restriction endonuclease digestion of the PCR-amplified product. Agarose gel electrophoresis was used to analyze the cleavage products. Following agarose gel electrophoresis, the cleavage products were detected after staining with Ethidium bromide under the UV light. The expected product sizes were 192 and 60 bp for the CC genotype, 252 bp for the TT genotype and products of 252, 192 and 60 bp for the heterozygous genotype (CT) genotype.

### *IL13* gene p.(Arg130Glu; rs20541) polymorphism

The *IL13* gene polymorphism *p*.(Arg130Glu; rs20541) was identified by PCR-RFLP method by the procedure described earlier (30, 31). This polymorphism results in an amino acid change in ‘exon 4 (G2044A), codon 130 in the *IL13* gene. Homozygous AA genotype codes for Glutamine (Q) and homozygous GG genotype encodes for Arginine (R) residue at codon 130. It is a common practice to use QQ and RR to represent the resulting amino acids from this polymorphism. The PCR products were cleaved with restriction enzyme *Nla*IV (0.5 U) at 37 °C for 3 h. The cleavage products were analyzed by Agarose gel electrophoresis and detected under UV light after staining with Ethidium bromide. The cleavage products were 210 bp and 26 bp in the case of AA (QQ) genotype and in individuals with GG (RR) genotype the product sizes obtained were 178 bp, 32 bp and 26 bp. The heterozygous individuals with AG (QR) genotype had the products with sizes, 210 bp, 178 bp, 32 bp and 26 bp respectively.

### Identification of HLA-DQ and DR genotypes

HLA-DQ and DR genotypes were identified by using a polymerase chain reaction-sequence specific primers method [PCR-SSP (32),]. DYNAL DQ and DR, PCR-SSP kits (Dynal, Oslo, Norway) were used to detect the genotypes in T1DM patients. The PCR conditions and subsequent analysis was carried out as described in the kit's Instructions manual. The genotypes were ascertained by using the “Interpretation Table” supplied with the kits.

### Statistical analysis

The data was analyzed using the Statistical Package for Social Sciences version 25 (SPSS, Chicago IL, United States). The frequencies of various genotypes and alleles detected in T1DM patients and controls were calculated by direct counting. The confidence interval (CI) was set at 95% and statistical significance was at *P* < 0.05 (two-tailed). The *P* values were corrected for multiple comparisons by Bonfferoni's correction. Fisher's Exact test was used to determine statistical significance of the differences between genotype and allele frequency between the T1DM patients and the controls. For calculation of the statistical significance in co-dominant and dominant genetic models, the genotype frequency in homozygous TT (*IL4* gene) and RR subjects (*IL13* gene) and the “T” and “R” allele frequency was considered as reference [as per previous reports (29–31)]. In the dominant model, genotype frequencies in respective heterozygous and homozygous subjects were pooled and were used in the statistical analysis. A posteriori power analysis was carried out to evaluate the strength of statistical analysis.

## Results

### Patient characteristics and clinical data

The characteristics of T1DM patients and controls are presented in Table 1. The Male/Female distribution was 128–133 in the T1DM patients' group while it was 110–104 in the controls (the difference is not statistically significant; *P* = 0.50; Table 1). The mean age of subjects in the T1DM patients' group was 115.05 ± 38.44 months and that of the control group was 115.18 ± 32.7 months respectively (the difference is not statistically significant). The average disease duration in the T1DM patients was <6 months. The age of onset of T1DM was below 4 years (y) in 54 (20%) patients; between 4–6y in 73 (28%) patients and >6y in 133 (52%) patients respectively (Table 1). The rate of consanguinity amongst parents of T1DM patients was 35% and in the controls it was 42% with no statistically significant difference (Table 1). The data on HbA1C at diagnosis and bone profile is also presented in Table 1. In all the control subjects the HbA1C was <5.7% while in 76% T1DM patients it was between 7%–10% and in 24% T1DM patients HbA1C was >10% (Table 1).

**Table 1:** Characteristics of Kuwaiti T1DM patients (*n* = 261) and controls (*n* = 214) included in the study.

	T1DM Patients	Controls	*P*-value*	OR**	95% CI**
**Gender**					
Males	128	110	0.50	0.84	0.575–1.241
Females	133	104	0.51	1.062	0.792–1.691
HbA1C (at diagnosis)***					
<5.7%	0	214 (100%)			
7%–10%	198 (76%)	0			
>10%	63 (24%)	0			
**Bone profile Ca ^± ±^ (mg/dl)**					
Range	1.8–2.7				
Mean	2.4				
PO_4_^−^					
Range	0.5–1.89				
Mean	1.38				
**Alkaline phosphatase (U/l)**					
<100	10 (4%)				
100–200	52 (20%)				
>200	198 (76%)				
**Consanguinity in parents**					
91 (35%)	90 (42%)			NS	
**Age at onset of T1DM (No. of subjects)**					
<4 y	54 (20%)				
4–6 y	73 (28%)				
>6 y	134 (52%)				

*P values were considered significant when < 0.05.

**OR, Odds ratio, 95% CI, 95% confidence interval; y (years).

***For screening of diabetes, HbA1C < 5.7% is considered absence of diabetes, 5.7–6.4% considered as increased risk for diabetes (prediabetes) >/= 6.5% is considered as consistent with diabetes (ISPAD Guidelines, 27); NS, not significant.

### Genotype and allele frequencies of *Il4* and *Il13* gene polymorphisms

**Figure 1:**
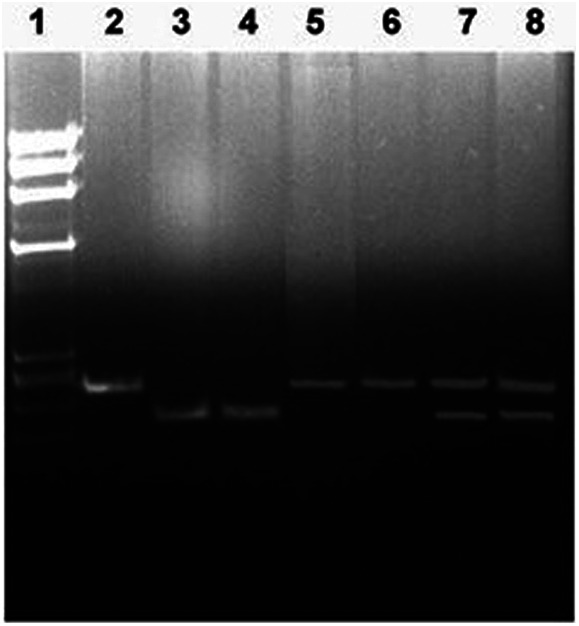
Detection of *IL4* gene (–C950T, rs2243250) polymorphism genotypes. Lane 1, *Hae*II cleaved *ϕ*X174 DNA *M_r_* size markers; lane 2, un-cleaved PCR product (252 bp); lane 3–4, PCR products cleaved with restriction enzyme *Bsm*F1 from subjects with CC genotype (192 bp); lanes 5–6, PCR products cleaved with restriction enzyme *Bsm*F1 from subjects with TT genotype (252 bp); lanes 7–8, PCR products cleaved with restriction enzyme *Bsm*F1 from subjects with CT genotype (252 and 192 bp). The restriction enzyme cleavage products were analyzed on 2% agarose gel and visualized under UV light after staining with Ethidium bromide.

**Figure 2:**
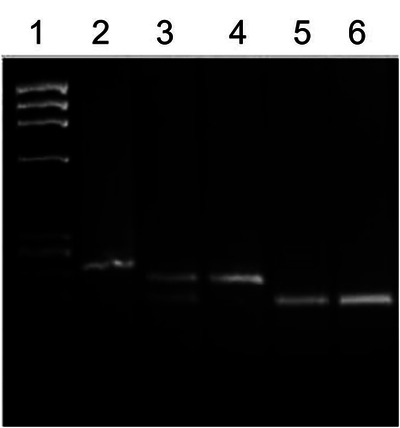
Detection of *IL13* gene polymorphism *p*.(Arg130Glu; rs20541) genotypes. Lane 1, *Hae*II cleaved *ϕ*X174 DNA *M_r_* size markers; lane 2, un-cleaved PCR product (210 bp); lane 3, PCR products cleaved with restriction enzyme *Nla*IV from a subject with RQ genotype (210 and 178 bp); lanes 4, PCR products cleaved with restriction enzyme *Nla*IV from a subject with RR genotype (210 bp); lanes 5-6, PCR products cleaved with restriction enzyme *Nla*IV from subjects with QQ genotype (178 bp). The restriction enzyme cleavage products were analyzed on 2% agarose gel and visualized under UV light after staining with Ethidium bromide.

The *IL4* gene (-C590T, rs2243250) polymorphism and *IL13* gene polymorphism *p*.(Arg130Glu; rs20541) were detected by PCR-RFLP method as described in Methods section. Examples of the typical results of these analyses are presented in Figures 1, 2 which show both the homozygous and heterozygous genotype patterns detected by these methods.

The frequencies of *IL4* gene -C590T polymorphism genotypes and alleles are presented in Table 2. The genotype frequency of *IL4* gene (-C590T) polymorphism (CC) was significantly higher in T1DM patients compared to that in the controls in Kuwaiti children (OR 1.64 and 1.24 respectively using the dominant and co-dominant models, Table 2). The frequency of “C” allele was also significantly higher in T1DM patients compared to that in the controls (OR 1.74, Table 2).

**Table 2:** Frequency of *IL4* gene -C590T polymorphism genotypes and alleles in Kuwaiti T1DM patients and controls.

Genotype/Alleles	Patients *N* = 244 (%)	Control *N* = 200 (%)	OR (95% CI)*	*P*-Value**
***Co-dominant***				
TT	18 (7.4)	18 (9.0)	1.00 (Reference)^a^	
CT	54 (22.1)	77 (38.5)	0.70 (0.33–1.47)	0.35
CC	172 (70.5)	105 (52.5)	1.64 (0.82–3.29)	**0**.**02**
***Dominant***				
TT	18 (7.4)	18 (9.0)	1.00 (Reference)^a^	
CT/CC (CT + CC)	226 (92.6)	182 (91.0)	1.24 (0.63–2.46)	**0**.**05**
***Alleles***	2N = 488	2N = 400		
T	90 (18.4)	113 (28.2)	1.00 (Reference)^a^	
C	398 (81.6)	287 (71.8)	1.74 (1.27–2.39)	**0**.**0007**

*OR (95% CI), Odds ratio, 95% confidence interval; **P-values were considered significant when < 0.05, shown in bold.

^a^ Genotype frequency in homozygous subjects with TT genotype and allele frequency of “T” allele were considered as reference for calculation of statistical significance using Fisher's Exact test. *N*, number of subjects included in each group. 2*N*, number of alleles derived from the N (the number of subjects) in each group.

In the case of *IL13* gene polymorphism *p*.(Arg130Glu; rs20541), statistically significant difference was detected in the frequency of homozygous QQ (AA) and heterozygous RQ (AG) genotypes (OR 4.79 and 2.92 in co-dominant model and OR 3.35 in the dominant model, Table 3). The “Q” allele frequency also showed a statistically significant difference between T1DM patients and the controls (*P* = 0.03, Table 3).

**Table 3:** Frequency of *IL13* gene *p*. (Arg130Glu; rs20541) genotypes and alleles in Kuwaiti T1DM patients and controls. The genotype AA of rs20541 polymorphism results in an Arginine (R) residue at codon 130 while genotype GG produced Glutamine (Q) amino acid shown as homozygous RR and QQ and heterozygous RQ.

Genotype/Alleles	Patients *N* = 244 (%)	Control *N* = 200 (%)	OR (95% CI)*	*P*-Value**
***Co-dominant***				
RR	3 (1.2)	8 (4.0)	1.00 (Reference)^a^	
RQ	79 (32.4)	44 (22.0)	4.79 (1.21–18.98)	**0**.**02**
QQ	162 (66.4)	148 (74.0)	2.92 (0.76–11.21)	**0**.**01**
***Dominant***				
RR	3 (1.2)	8 (4.0)	1.00 (Reference)^a^	
RQ/QQ (RQ + QQ)	241 (98.8)	192 (96.0)	3.35 (0.88–12.79)	**0**.**07**
***Alleles***	2N = 488	2N = 400		
R	85 (17.4)	60 (15.0)	1.00 (Reference)^a^	
Q	403 (82.6)	340 (85.0)	0.84 (0.58–1.20)	**0**.**03**

*OR (95% CI), odds ratio 95% confidence interval; **P-values were considered significant when < 0.05, shown in bold.

^a^ Genotype frequency in homozygous subjects with RR genotype and allele frequency of “R” allele were considered as reference for calculation of statistical significance using Fisher's Exact test. *N*, number of subjects included in each group. 2*N*, number of alleles derived from the *N* (the number of subjects) in each group.

### Findings on HLA-DQ and DR genotypes in Kuwaiti T1DM patients

The frequency of HLA-DQ genotypes detected in Kuwaiti T1DM patients and controls is presented in Table 4. Altogether, nineteen different genotypes were detected. Six of these (DQ1/2; DQ2/2, DQ2/3, DQ2/5, DQ2/8 and DQ8/8) showed a statistically significant association with T1DM compared to the controls (Table 4). The highest association between DQ genotypes and T1DM was detected in DQ1/DQ2 (OR 28.24, *P* < 0.0001), DQ2/DQ2 (OR 7.73, *P* < 0.0001), DQ2/DQ8 (OR 20.99, *P* < 0.0001) and DQ8/DQ8 (OR 2.47, *P* < 0.05) respectively (Table 4). In 98 (55%) T1DM patients, the genotype was either homozygous for DQ2 or in combination with a DQ8 allele (Table 4). Similarly, in 58 (36%) patients, the genotype was homozygous for DQ8 or with other alleles. Altogether, 91% of the T1DM patients had either DQ2 or DQ8 alleles in different combinations with other alleles (Table 4). The frequency of HLA-DR genotypes detected in T1DM patients and controls is presented in Table 5. A statistically significant association was detected in the case of DR3/DRB5 (OR 7.73, *P* 0.003), DR3/DR4 (OR 32.77, *P* < 0.0001), DR3/DR7 (OR 19.41, *P* = 0.003) and DR4/DR4 (OR 14.50, *P* 0.01) respectively (Table 5).

**Table 4:** Frequency of HLA DQ genotypes in Kuwaiti T1DM patients and controls. The genotypes were determined by using PCR-SSP method (Dynal kits, Oslo, Norway) as described in Materials and Methods.

Genotype	T1DM patients *N* = 217	Controls *N* = 184	OR*	*P*- value**
DQ1/DQ1	1	0	2.56 (0.10–63.19)	1.00
**DQ1/DQ2**	15	0	28.24 (1.68–48.71)	<0.0001
DQ1/DQ8	3	3	0.85 (0.17–4.24)	1.00
**DQ2/DQ2**	62	9	7.78 (3.74–16.17)	<0.0001
**DQ2/DQ3**	14	3	4.16 (1.18–14.78)	0.03
**DQ2/DQ5**	22	4	5.05 (1.72–15.02)	0.001
DQ2/DQ6	1	0	2.55 (0.10–63.18)	1.00
DQ2/DQ7	7	2	3.03 (0.62–14.79)	0.19
**DQ2/DQ8**	56	3	20.99 (6.44–68.37)	<0.0001
DQ3/DQ3	2	28	0.05 (0.01–0.22)	<0.0001
DQ3/DQ7	1	31	0.023 (0.00–0.17)	<0.0001
DQ3/DQ8	2	5	0.33 (0.63–1.74)	0.26
DQ4/DQ8	1	2	0.42 (0.04–4.69)	0.60
DQ5/DQ5	3	38	0.05 (0.02–0.18)	<0.0001
DQ5/DQ8	6	2	2.58 (0.56–12.98)	0.29
DQ6/DQ8	5	1	4.32 (0.49–37.30)	0.23
DQ7/DQ7	1	28	0.03 (0.00–0.19)	<0.0001
**DQ8/DQ8**	14	5	2.47 (0.87–6.99)	0.01
DQ9/DQ9	1	20	0.04 (0.01–0.29)	<0.0001

*OR (95% CI), Odds ratio, 95% confidence interval;.

**P-values were considered significant when < 0.05, Genotypes shown in bold show a significant association with T1DM. *N*, the number of subjects in which genotypes were identified in each group.

**Table 5:** Frequency of HLA DR genotypes in Kuwaiti T1DM patients and controls. The genotypes were determined by using PCR-SSP method (Dynal kits, Oslo, Norway) as described in Materials and Methods.

Genotype	T1DM patients *N* = 92	Controls *N* = 96	OR*	*P*-value**
DR1/DRB3	2	1	2.11 (0.18–23.70)	0.62
DR3/DRB4	1	1	1.04 (0.06–16.95)	1.00
**DR3/DRB5**	13	2	7.73 (1.69–35.32)	0.003*
DR7/DRB4	1	3	0.34 (0.04–3.34)	0.62
DRB3/DR6	3	1	3.20 (0.33–31.37)	0.36
DRB3/DR6	3	1	3.20 (0.33–31.37)	0.36
DRB3/DR7	4	1	4.32 (0.47–39.40)	0.20
DRB3/DRB4	5	1	5,046 (0.63–47.68)	0.11
DRB3/DRB5	1	0	3.16 (0.13–78.72)	0.49
DRB4/DRB5	2	8	0.24 (0.05–1.18)	0.10
DRB4-DR7	1	7	0.14 (0.02–1.16)	0.06
DR4/DR7	1	5	0.20 (0.02–1.75)	0.21
DR2/DR2	1	18	0.05 (0.01–0.37)	<0.0001*
DR2/DR3	1	1	1.04 (0.06–16.95)	1.00
DR3/DR3	7	2	3.87 (0.78–19.15)	0.09
**DR3/DR4**	13	0	32.77 (1.92–56.35)	<0.0001*
DR3/DR6	2	0	5.33 (0.25–11.65)	0.24
**DR3/DR7**	8	0	19.41 (1.10–34.65)	0.003*
DR3/DR8	1	1	1.04 (0.06–16.95)	1.00
DR3/DR11	1	1	1.04 (0.06–16.95)	1.00
DR3/DR16	3	1	3.20 (0.33–31.37)	0.36
**DR4/DR4**	6	0	14.50 (0.80–26.41)	0.01*
DR4/DR6	2	0	5.33 (0.25–11.65)	0.24
DR4/DR7	4	0	9.81 (0.52–18.02)	0.06
DR4/DR11	3	0	7.55 (0.38–14.27)	0.12
DR4/DR16	1	0	3.16 (0.13–78.72)	0.49
DR7/DR11	1	23	0.04 (0.01–0.26)	<0.0001*
DR9/DR9	1	18	0.05 (0.01–0.37)	<0.0001*

*OR (95% CI), Odds ratio, 95% confidence interval.

**P-values were considered significant when < 0.05, Genotypes in bold show a significant association with T1DM. *N*, the number of subjects in which genotypes were identified in each group.

### Co-inheritance of *Il4* gene (–C590T, rs2243250) polymorphism genotypes with HLA-DQ genotypes in Kuwaiti T1DM patients

**Figure 3:**
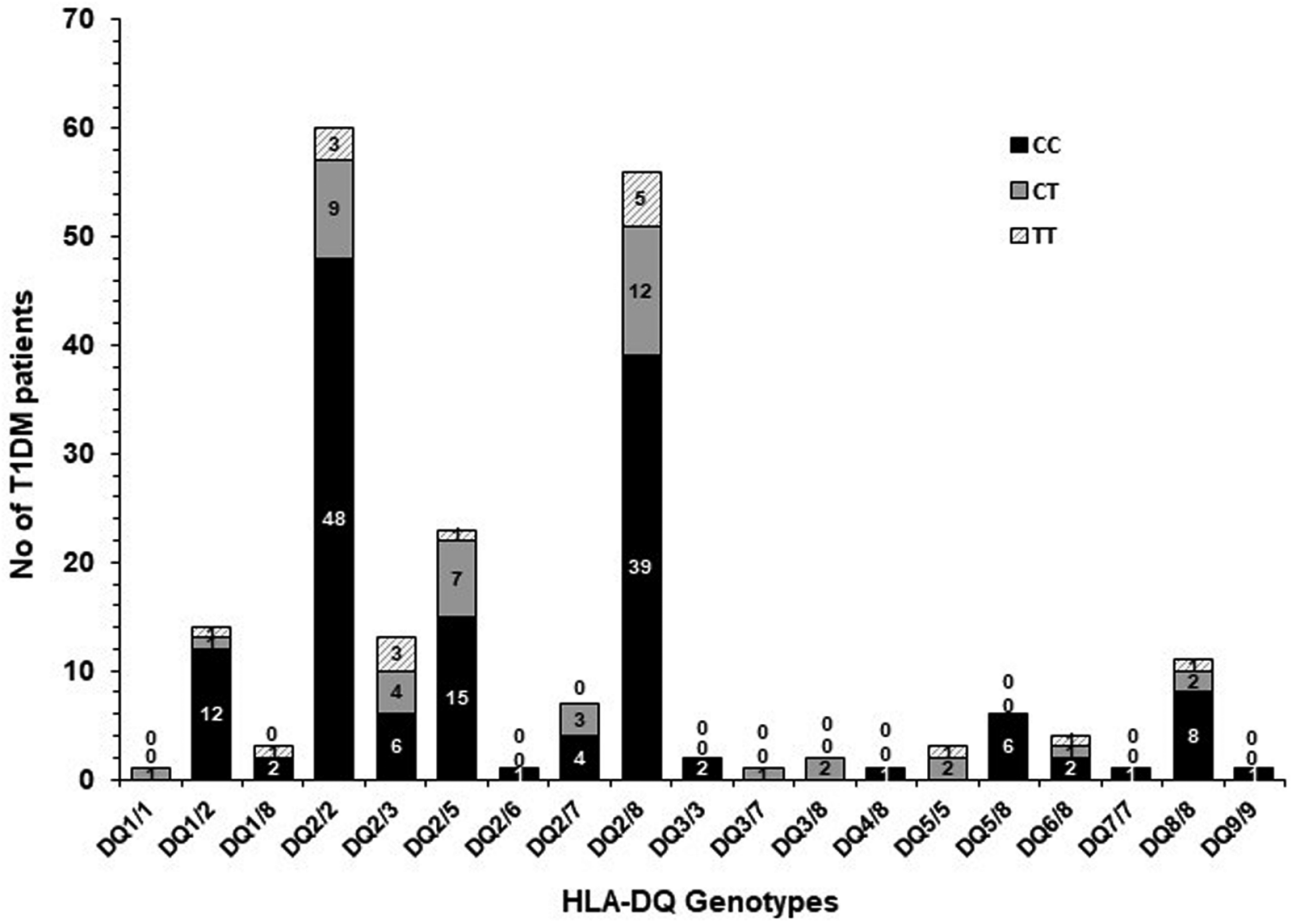
Co-inheritance of *IL4* gene –C590T polymorphism genotypes with HLA-DQ genotypes in Kuwaiti T1DM patients. The shaded bars show the co-inheritance with various HLA-DQ genotypes.

The frequency of co-inheritance of *IL4* gene (–C590T, rs2243250) polymorphism genotypes with HLA-DQ genotypes in Kuwaiti T1DM patients is presented in Figure 3. In Kuwaiti T1DM patients with high incidence/high risk HLA-DQ2/DQ2 genotype, the majority (48/60, 80%) carried “CC” genotype of the *IL4* gene polymorphism. When the homozygous (CC) and heterozygous (CT) genotypes (containing at least one C-allele) were considered together, they were detected in 95% of the T1DM patients with high risk DQ2/DQ2 genotype (Figure 3). In Kuwaiti T1DM patients who carried the other high-risk HLA-DQ2/DQ8 genotype, 39/56 (70%) had the homozygous CC genotype and 87% co-inherited at least one variant “C” allele of the *IL4* gene polymorphism along with the high-risk HLA-DQ2/DQ8 genotype (Figure 3).

### Co-inheritance of *Il4* gene (–C590T, rs2243250) polymorphism genotypes with HLA-DR genotypes in Kuwaiti T1DM patients

**Figure 4:**
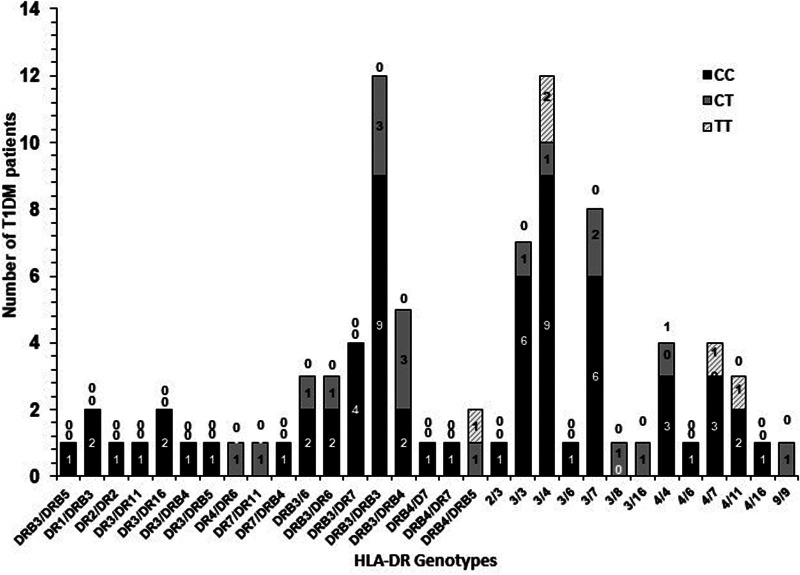
Co-inheritance of *IL4* gene –C590T polymorphism genotypes with HLA-DR genotypes in Kuwaiti T1DM patients. “0” above the bars indicate the absence of a particular genotype. The shaded bars show the co-inheritance with various HLA-DR genotypes.

The frequency of co-inheritance of *IL4* gene (–C590T, rs2243250) polymorphism with HLA-DR genotypes in Kuwaiti T1DM patients is presented in Figure 4. It is interesting to note that amongst the Kuwaiti T1DM patients who carried the high-risk HLA-DR genotypes (DR3/DRB5, DR3/DR4, DR3/DR7, DR4/DR4), the “C” allele (in homozygous and heterozygous CT combinations) is detected in majority of the patients (Figure 4).

### Co-inheritance of *Il13* gene p.(Arg130Glu; rs20541) polymorphism genotypes with HLA-DQ genotypes in Kuwaiti T1DM patients

**Figure 5:**
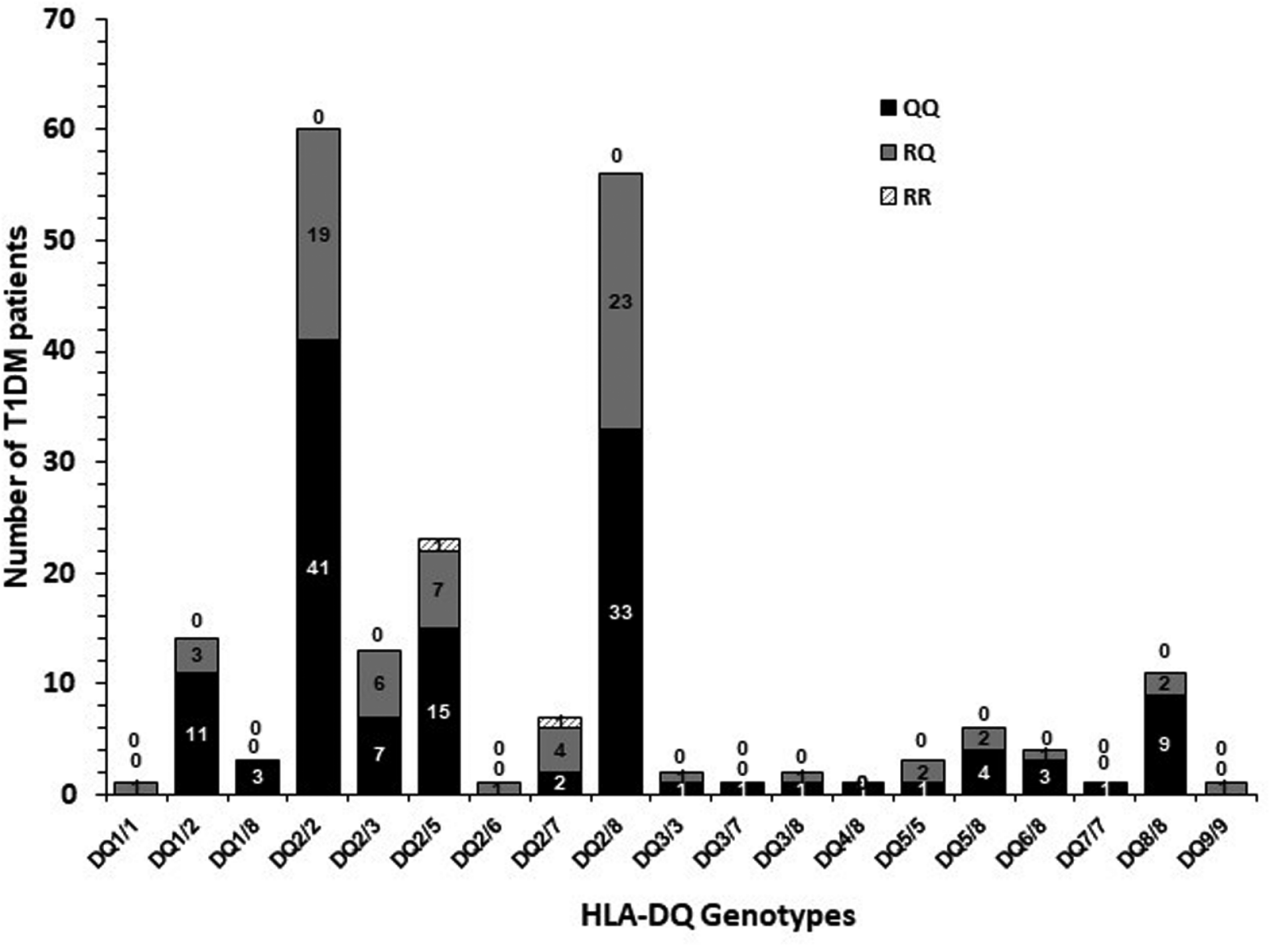
Co-inheritance of *IL13* gene *p*.(Arg130Glu; rs20541) polymorphism genotypes with HLA-DQ genotypes in Kuwaiti T1DM patients. “0” above the bars indicate the absence of a particular genotype. The shaded bars show the co-inheritance with various HLA-DQ genotypes.

The frequency of co-inheritance of *IL13* gene *p*.(Arg130Glu; rs20541) polymorphism with HLA-DQ genotypes in Kuwaiti T1DM patients is presented in Figure 5. In Kuwaiti T1DM patients who carried the high-risk HLA DQ2/DQ2 and DQ2/DQ8 genotypes, the co-inheritance of the “Q” allele was in 100% patients (Figure 5).

### Co-inheritance of *Il13* gene p.(Arg130Glu; rs20541) polymorphism genotypes with HLA-DR genotypes in Kuwaiti T1DM patients

**Figure 6:**
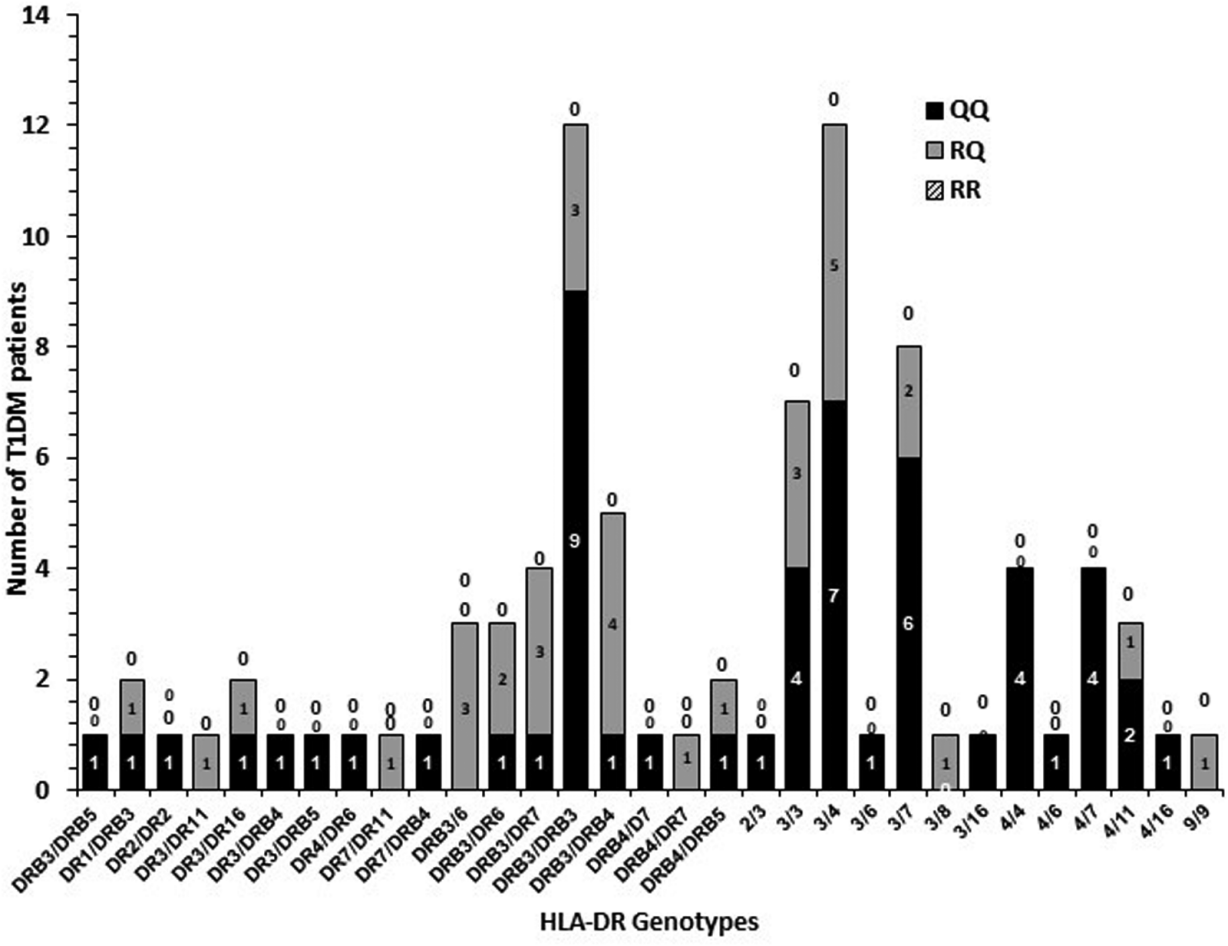
Co-inheritance of *IL13* gene *p*.(Arg130Glu; rs20541) polymorphism genotypes with HLA-DR genotypes in Kuwaiti T1DM patients. “0” above the bars indicate the absence of a particular genotype. The shaded bars show the co-inheritance with various HLA-DR genotypes.

The frequency of co-inheritance of *IL13* gene *p*. (Arg130Glu; rs20541) polymorphism with HLA-DR genotypes in Kuwaiti T1DM patients is presented in Figure 6. A very high co-inheritance between “Q” (A) allele and all the high-risk HLA-DR genotypes (DR3/DRB5, DR3/DR4, DR3/DR7 and DR4/DR4), was detected (Figure 6).

## Discussion

Our findings demonstrate a strong and significant association between the CC genotype of *IL4* gene (−C590T, rs2243250) polymorphism in Kuwaiti T1DM patients. This is in sharp contrast to the data from Saudi Arabian population (33), in which the authors have reported that the Saudi individuals with homozygous TT genotype of the *IL4* gene (C590T, rs2243250) polymorphism were significantly more susceptible to develop T1DM (OR 2.23; *P* < 0.001). The heterozygous (CT) genotype was not significantly associated with T1DM in the study from Saudi Arabia (33). This can be attributed to a dose effect in heterozygous combination where only one copy of the susceptibility allele is present and may not produce its product in sufficient amount to manifest the susceptibility. A study from Filipinos, reported that the risk for T1DM is determined, in part, by polymorphisms within the *IL4-R* locus, including promoter and coding-sequence variants, and by specific combinations of genotypes at the *IL-R* and the *IL4* and *IL13* loci (34). However, soon after its publication, these findings were refuted by a much larger study from United Kingdom and United States populations [Caucasians (35),]. This report from Caucasians did not find any single-locus *IL4* and *IL13* gene associations with T1DM and could not find any evidence of gene-gene interaction as reported in Filipinos (35). A meta-analysis describing the relationship between *IL4* gene promoter (−C590T; rs2243250) polymorphism and susceptibility to autoimmune diseases was published in 2014 (15). This meta-analysis reported that *IL4* rs2243250 polymorphism might be associated with genetic susceptibility of autoimmune diseases including RA and MS (15). It is interesting to note that in this meta-analysis, it was the “C” allele of the *IL4* gene (–C590T, rs2243250) polymorphism, which was found to be associated with RA and MS. However, in that meta-analysis, no association was reported in the case of SLE or GD (15). Our findings from Kuwaiti children with T1DM are similar to these i.e., the CC genotype of *IL4* gene polymorphism manifest a strong and significant association with T1DM in a high prevalence population (Kuwaiti children, Table 2).

Another interesting finding in this study is that a significant association was detected between the AA (Q) genotype and “Q” allele of *IL13* gene *p*.(Arg130Glu) polymorphism and T1DM in Kuwaiti children (Table 3). The potential role of anti-inflammatory cytokines e.g., IL4 and IL13 in the etiology of T1DM has received attention recently. There is evidence to suggest that these molecules have a significant impact on the beta-cell function and viability (36). IL4 has been shown to promote humoral immunity and mediate anti-inflammatory effects such as inhibition of proinflammatory cytokines, regulation of anti-inflammatory and anti-apoptotic genes (36). It has been shown in NOD mice that ectopic expression of IL4 in beta-cells prevented infiltration of CD8+ T cells and resulted in reducing the incidence of T1DM (37). Also, treatment of NOD mice with recombinant IL4 resulted in protection from diabetes (38). It has also been demonstrated that exposure of human beta-cell lines to IL4 decreased cell death when exposed to pro-inflammatory cytokines by activation of the STAT6 pathway (39, 40). Recent evidence has shown that NOD mice lacking the heteroreceptor for IL4 and IL13 display resistance to T1DM thus supporting the hypothesis that physiological levels of IL4/IL13 (which are correlated with gene polymorphisms such as *IL4* gene -C590T, rs2243250 and *IL13* gene *p*.(Arg130Glu) may contribute to the onset of T1DM (41).

A report from the Dutch population investigated the involvement of several Th1, Th2 cytokine genes and the metabolic genes in genetic susceptibility of T1DM (42). In this report, although none of the individual polymorphism was found to be transmitted more frequently than expected, it was revealed that additional genetic predisposition of T1DM is defined by combinations of markers (e. g. Th2 and metabolic) rather than by a single marker (42). Based on studies in Dutch population, it was postulated that the consequences of increased transmission of a low Th2 expressing genotype (e. g. *IL4* gene CC) together with a normal Th1 profile may result in a net pro-inflammatory cytokine expression pattern (42). In other words, it was suggested that the Th1 disease like T1DM, is not influenced by a genetic predisposition involving the Th1 genes, but by a distortion of the Th2 and metabolic gene cluster (42). They further postulated that owing to the extreme sensitivity of the Th1-Th2 balance, even a slight decrease in the Th2 cytokines (for example, that is expected in T1DM patients with *IL4* gene CC genotype), will result in a relative Th1 cytokine overload, possibly resulting in destructive insulitis (37). Our results from Kuwaiti T1DM children (higher CC genotypes in T1DM patients, Table 2), corroborate these suggestions and findings from Dutch patients reported earlier (42). Eerligh et al. (42) have proposed a “Multiple-Hit” theory to explain the genetic involvement in pathogenesis of T1DM. According to this theory, islet beta-cell destruction may result from an interaction of many if not all, of the products from genes involved in the pathogenesis of T1DM; it is conceivable that the “candidate genes” interact! This theory further states that a single locus may harbor an undetectable small contribution to the “relative risk” and only when combinations of genes (synergistically involved in the same regulatory pathway) are analyzed together, the “relative risk” may increase to a detectable level. Our data on the co-inheritance of “High-risk” HLA-DQ and DR genotypes with the *IL4* and *IL13* gene polymorphism genotypes supports the “Multiple-Hit” concept in determining genetic susceptibility of the T1DM in Kuwaiti children.

## Conclusions

In this study, we have identified *IL4* gene polymorphism genotype (CC) and *IL13* gene polymorphism genotype (AA/Q) as high-risk genotypes in the Kuwaiti T1DM patients. This study also report a significant co-inheritance of the CC genotype of *IL4* gene polymorphism (−590C/T, rs2243250), and the (AA/Q) genotype of *IL13 p*.(Arg130Glu) gene polymorphism with the high-risk HLA-DQ and DR genotypes and highlights their significant role in genetic susceptibility of the T1DM in Kuwaiti Arab children.

## Data Availability

The original contributions presented in the study are included in the article/Supplementary Material, further inquiries can be directed to the corresponding author.
